# Key factor cutoffs and interval reference values for stratified fall risk assessment in community-dwelling older adults: the role of physical fitness, body composition, physical activity, health condition, and environmental hazards

**DOI:** 10.1186/s12889-021-10947-x

**Published:** 2021-11-10

**Authors:** Catarina Pereira, Guida Veiga, Gabriela Almeida, Ana Rita Matias, Ana Cruz-Ferreira, Felismina Mendes, Jorge Bravo

**Affiliations:** 1 Departamento de Desporto e Saúde, Escola de Saúde e Desenvolvimento Humano, Universidade de Évora, Largo dos Colegiais 2, Évora, Portugal; 2 Comprehensive Health Research Centre (CHRC), Universidade de Évora, Largo dos Colegiais 2, Évora, Portugal; 3 Escola Superior de Enfermagem São João de Deus, Universidade de Évora, Largo do Sr. da Pobreza 2B, Évora, Portugal

**Keywords:** Falling, Elderly, Risk assessment, Cutoffs, Risk stratification

## Abstract

**Background:**

Fall risk assessment and determination of older adults’ individual risk profiles are crucial elements in fall prevention. As such, it is essential to establish cutoffs and reference values for high and low risk according to key risk factor outcomes. This study main objective was to determine the key physical fitness, body composition, physical activity, health condition and environmental hazard risk outcome cutoffs and interval reference values for stratified fall risk assessment in community-dwelling older adults.

**Methods:**

Five-hundred community-dwelling Portuguese older adults (72.2 ± 5.4 years) were assessed for falls, physical fitness, body composition, physical (in) activity, number of health conditions and environmental hazards, and sociodemographic characteristics.

**Results:**

The established key outcomes and respective cutoffs and reference values used for fall risk stratification were multidimensional balance (low risk: score > 33, moderate risk: score 32–33, high risk: score 30–31, and very high: score < 30); lean body mass (low risk: > 44 kg, moderate risk: 42–44 kg, high risk: 39–41 kg, and very high: < 39 kg); fat body mass (low risk: < 37%, moderate risk: 37–38%, high risk: 39–42%, and very high: > 42%); total physical activity (low risk: > 2800 Met-min/wk., moderate risk: 2300–2800 Met-min/wk., high risk: 1900–2300 Met-min/wk., and very high: < 1900 Met-min/wk); rest period weekdays (low risk: < 4 h/day, moderate risk: 4–4.4 h/day, high risk: 4.5–5 h/day, and very high: > 5 h/day); health conditions (low risk: *n* < 3, moderate risk: *n* = 3, high risk: *n* = 4–5, and very high: *n* > 5); and environmental hazards (low risk: *n* < 5, moderate risk: *n* = 5, high risk: *n* = 6–8, and very high: *n* > 8).

**Conclusions:**

Assessment of community-dwelling older adults’ fall risk should focus on the above outcomes to establish individual older adults’ fall risk profiles. Moreover, the design of fall prevention interventions should manage a person’s identified risks and take into account the determined cutoffs and respective interval values for fall risk stratification.

## Background

Falls are a problem for older adults, caregivers, society, governors, and world health organizations [1, 2]. Falling causes pain, injuries, disability, dependence, deaths, and long-term psychological harm [3], which, in turn, have a very high economic cost [2, 4]. Therefore, the design of effective strategies for fall prevention interventions is a global concern.

Fall risk assessment has been identified as a crucial element in fall prevention; most likely, such assessment enables the identification of older adults who are at high risk of falling, thereby allowing the person to participate in a fall prevention program [5]. Such assessment is particularly important because many older adults lack the perception of their own fall risk [6], as do some caregivers [7]. The American Geriatric Society and the Centers for Disease Control and Prevention recommend screening for fall risk in older adults at least annually and emphasize that this screening should address modifiable fall risk factors [8]. The screening may also serve to measure and rank a person’s risk of falling, although there is not a consensus on the definition of high- low risk [9].

Most recent studies focusing on fall risk assessment involve software, multimedia devices, and other technological apparatuses [10, 11]. However, traditional approaches comprising field tests and questionnaires (e.g., *Senior Fitness Test, Fullerton Advanced Balance Scale*, *International Physical Activity Questionnaire)* remain very useful, especially considering that they are easy, low cost, and friendly instruments for older adults [12, 13]. The most reported risk factors for falls are cognitive impairment, physical fitness (strength, flexibility, agility, balance, aerobic endurance, etc.), gait, body composition (e.g., lean and fat mass), physical activity (e.g., light, moderated or vigorous), health condition and environmental hazards [14]. However, to date, it is not clear which risk factors are the key risk factors for falling and which key measures or outcomes should be considered and evaluated to determine the individual fall risk profiles of older adults. Indeed, to be effective, fall prevention programs should be focused on factors that determine falls (key risk factors) and are present and impaired in each older adult at risk of falling [14–16].

In addition, it is critical to know the cutoffs of each usable key factor outcome to establish reference range values associated with a low-high risk of falling. Several studies have established cutoffs of risk for some factors. For example, Hernandez and Rose [17] established a cutoff for the single risk factor multidimensional balance; however, the determination of older adults’ fall risk profile requires a multifactorial approach. Although Nithman and Vincenzo [18] developed the STEADI toolkit, including several key risk factors (i.e., previous falls, gait, strength, and gait) and the respective cutoffs for fall risk screening, some important factors were excluded. In fact, the literature [18, 19] shows not only that some interactions between risk factors were not considered in previous research but also that some key factors were excluded from fall risk stratification, such as fat body mass, day rest period and environmental hazards. Moreover, in other cases, the stratification process (high-moderate-low) was not completed, as a single cutoff point was established to distinguish fallers from nonfallers [17].

The establishment of risk range (high-low) reference values for all key risk factor outcomes will enable comparison of the results regarding each key risk factor test with respect to the reference values, allowing the easy establishment of the individual fall risk profile. This approach may be used to identify the key factors that contribute to fall risk and quantify their deviation from safe values. This knowledge will also alert older adults and their caregivers for the need to attend a fall prevention program. It will also allow physicians and other health professionals to outline highly targeted and effective fall prevention interventions. Thus, this study’s main objective was to determine the key physical fitness, body composition, physical activity, health condition, and environmental hazard risk factor outcome cutoffs and interval reference values for a stratified fall risk assessment in community-dwelling older adults.

## Methods

### Participants

Participants were independent community-dwelling Portuguese older adults aged 65 years and over. They were enrolled in the study via leaflets and posters distributed in community settings (e.g., health centers, recreational, sports and cultural associations, universities for seniors). The sample size was estimated by the online OpenEpi software as 384 (http://www.openepi.com/SampleSize/SSCohort.htm), keeping the confidence interval (CI) at 95% and the level of significance at 5%. Eligible older adults were 517. Inclusion criteria required the absence of cognitive impairment following the Folstein Mini-Mental State Examination, with a cutoff of 24 points and below indicating a cognitive decline [20, 21]. Subjects who had suffered a recent health condition resulting in a temporary loss of physical fitness or dependence were excluded from the study. Hence, 17 respondents were excluded: ten were aged less than 65 years, three showed a cognitive decline, and three had suffered recent health conditions resulting in a temporary loss of physical fitness or dependence (one had a myocardial infarction in the last month, one had a hip fracture in the previous month, and one had a knee dislocation 2 months earlier). A total of 500 persons (362 women and 138 men) aged 72.2 ± 5.4 years, with 5.2 ± 3.9 years of school participated in the study, of which 37.2% had fallen at least once in the previous 12 months. All subjects were volunteers and provided written informed consent. This study was approved by the Ethics Committee of human health and well-being (16–012) following the Declaration of Helsinki.

### Outcome measures

The measures were undertaken in community settings, ensuring good evaluation conditions, individual attention, and participant privacy. A specialized team of experts - with an academic degree in nursing, sports, or rehabilitation sciences - attended a course on the published protocols and carried out the evaluations. Each questionnaire or test was always performed by the same evaluator, who was blind to the study’s objectives. The test-retest reliability (with a one-week interval between test and retest performed by the same evaluator) was calculated using the bivariate correlation of Spearman or Pearson [22] and ranged between 0.722 and 0.999. As many participants had never attended school, a single interviewer completed the questionnaires for all participants.

#### Falls

The occurrence of falls in the previous 12 months and the circumstances surrounding each fall (such as the reason for the fall, the location where the fall occurred, the action involved, and the consequences of the fall) were meticulously assessed by the interviewer using a questionnaire. Falls were defined as “*an unexpected event in which the participants come to rest on the ground, floor, or lower level*”, a faller was defined as a subject who had fallen at least once in the previous 12 months, and a nonfaller was defined as a subject who had not fallen in this period [23]. For statistical analyses, the faller condition was expressed as “1”, and the nonfaller condition was expressed as “0”.

#### Physical fitness and body composition

Multidimensional balance was assessed by the Fullerton Advanced Balance (FAB) scale [22]. The final score (0 to 40 points) was obtained by the sum of points obtained in each of the 10 FAB tests, each ranging from 0 (worst) to 4 (best). Lower and upper body strength (number of repetitions/30 s), lower and upper flexibility (cm), agility and dynamic balance (sec), aerobic endurance (m), and body mass index (kg/m^2^) were assessed using the Senior Fitness Test battery [24]. Standing height (cm) was measured with a stadiometer (Seca 770, Hamburg, Germany), and weight (kg) was measured using an electronic scale (Seca Bella 840, Hamburg, Germany). Fat (%) and lean body mass (kg) were assessed by bioimpedance [25] (Omron HBF-511 BE, USA).

#### Physical activity

Habitual physical activity was assessed using the short version of the International Physical Activity Questionnaire (IPAQ) [26] queried out by the interviewer. IPAQ covers metabolic expenditure (metabolic equivalent of task [MET] – min/wk) on walking (3.3 MET), moderate activity (4.0 MET), and vigorous activity (8.0 MET). The metabolic expenditure (MET min/wk) was calculated by determining the time (min/d) and frequency (d/wk) spent in each of these activities, and total physical activity was computed by the sum of their respective metabolic expenditures. For some data analyses, physical activity units were expressed as 100 MET-min/wk. The rest period weekdays (hr/day) - in addition to night sleep time - was also assessed as time spent seated (mean of the typical weekend and weekday).

#### Health conditions

Chronic diseases and physical impairments were assessed using a questionnaire queried out by the interviewer. Among all participants, 26 chronic diseases were listed. Physical impairments included involuntary loss of urine, frequent dizziness, foot problems, poor vision, hearing problems, and occasional loss of balance. The presence or absence of each of the listed chronic diseases and physical impairments was checked for each participant. The variable health conditions concerned the sum of the number of chronic diseases and physical impairments [27].

#### Environmental hazards

Environmental hazards included indoor hazards (lousy lighting, slippery floors, loose rugs, telephone cables, other objects, ladders, stairways with steep steps, without walls and/or handrails, kitchens with difficult access to utensils and movable tables, bathrooms without tub handrails, shower and toilet and nonskid mat in tub or shower, bed too high or too low), outdoor hazards (lousy lighting, uneven pavements, streets, paths, repair works, obstacles, slippery floors), the presence of animals, and footwear. The presence of each listed environmental hazard was checked for each participant, and the total number of hazards was counted (minimum: 0, maximum: 34) [28].

#### Sociodemographic characteristics

Sex, chronologic age (yr.) and education (yr.) were assessed by questionnaire.

### Data analysis

An exploratory analysis was performed to characterize the data and to assess the potential of each risk factor to explain fall occurrence by using univariate binary logistic regression. The percentiles for each potential risk factor for falling, as well as univariate odds ratios (ORs), were computed. For physical activity OR computation, units were 100 MET- min/wk.; therefore, the results are presented rounded up to the nearest hundred unit (i.e.: 18.87 units correspond to 1900 MET- min/wk).

Multivariate binary logistic regression analysis and receiver operating characteristic (ROC) analysis were used to select the key risk factors for falling (and respective evaluation outcomes), to quantify the implication of the variation of each key factor on fall occurrence risk (probability) and to establish the cutoffs of risk in each of these key factors’ outcomes [29]. For this aim, first, the fittest and most parsimonious multivariate binary logistic regression models (including physical fitness, body composition, physical activity, health condition and environmental hazards variables) were determined by using the traditional approach (a similar approach was performed by Almeida et al. [30]). The overall fit of the model was evaluated using the Hosmer-Lemeshow goodness-of-fit test. Second, ROC analysis, based on the area under the curve (AUC), was used to examine the built model’s ability to discriminate fallers from nonfallers. Third, the cutoff point for the probability of falling (π) was established by maximizing both sensitivity and specificity. The cutoff points of π: 0.25 and of π: 0.5 were also considered to stratify the risk of falling as low, moderate, high and very high; such that, low risk: π: < 0.25; moderate risk: 0.25 ≤ π:< cutoff which maximizes both sensitivity and specificity; high risk: cutoff which maximizes both sensitivity and specificity ≤ π: < 0.50; and very high risk: π: ≥ 0.50). Fourth, the outlined π cutoff points were used to compute each key factor percentile (value), which matches the above risk of falling stratification. For that, the regression equation was solved successively using the key factor outcome values from the 1st to 99th percentiles. Thus, the key factor outcome values that equated the falling probability cutoff points described above were identified as the cutoff values for each key factor’s outcomes (point estimation) usable for the risk level stratification (as low, moderate, high, or very high). Finally, the internal validation of the model was tested using a resampling or cross-validation procedure [31]. For this, participants were clustered into 10 equal groups by random sampling without replacement, and the AUC was calculated using the probabilities generated by cross-validation.

Statistical analyses were performed using the SPSS software package (Version 24.0 for Mac; SPSS, Chicago, IL, USA). Statistical significance was set as *p* < 0.05.

## Results

### Characterization and exploratory results

Table 1 shows the participants’ characteristics regarding potential risk factor outcomes for fall occurrence. The analysis of participants’ results expressed as percentiles evidence that there is a high variability of results in the different potential risk factors for falls among all participants. Results from univariate binary logistic regression shown that most study variables significantly explain fall occurrence individually (*p* < 0.05). The exceptions were lower-body flexibility, body weight, body mass index, lean body mass, walking physical activity, moderate physical activity, and total physical activity. Increasing lower and upper body strength, upper body flexibility, aerobic endurance, body height, and multidimensional balance decreased the likelihood of falling. Increasing the time spent on agility and dynamic balance test, body fat mass, vigorous physical activity, weekday rest periods, health conditions and environmental hazards increased the likelihood of falling (*p* < 0.05).

**Table 1:** Participants` characteristics regarding potential risk factors for fall occurrence (*n* = 500)

	1st P	25th P	50th P	75th P	99th P	OR (95%CI)
Lower body strength (rep)	7	13	15	18	30	0.965 (0.954–0.976)
Upper body strength (rep)	6	13	17	20	29	0.969 (0.959–0.980)
Lower body flexibility (cm)	−32.0	−8.0	0.0	3.0	18.5	1.010 (0.991–1.029)
Upper body flexibility (cm)	−37.0	−18.0	−10.0	−2.0	13.0	0.975 (0.959–0.992)
Agility and dynamic balance (sec)	3.9	5.1	5.7	6.6	12.2	1.182 (1.049–1.331)
Aerobic endurance (m)	240	438	499	550	714	0.997 (0.995–0.999)
Multidimensional balance score (point)	12	28	32	35	40	0.939 (0.910–0.968)
Body weight (kg)	44.9	61.0	68.6	77.4	108.1	0.997 (0.983–1.012)
Body height (cm)	137.0	150.0	155.0	162.9	176.0	0.969 (0.948–0.990)
Body mass index (kg/m^2^)	20.5	25.6	28.0	31.1	39.6	1.037 (0.993–1.082)
Lean body mass (kg)	26.3	37.1	42.2	48.9	67.9	0.982 (0.962–1.002)
Fat body mass (%)	15.5	32.6	38.3	43.3	48.5	1.037 (1.011–1.064)
Walking physical activity (MET-min/wk)	0	297	578	990	2772	0.970 (0.938–1.003) ^a^
Moderate physical activity (MET-min/wk)	0	960	1680	2400	10,080	0.992 (0.981–1.002) ^a^
Vigorous physical activity (MET-min/wk)	0	0	0	0	7200	1.015 (1.000–1.030) ^a^
Total physical activity (MET-min/wk)	170	1646	2473	3884	12,797	0.998 (0.990–1.006) ^a^
Rest period weekdays (hr/day)	0.6	3.0	4.3	5.5	10.0	1.129 (1.021–1.247)
Health conditions (n)	0	2	3	5	12	1.167 (1.088–1.251)
Environmental hazards (n)	0	4	6	9	21	1.057 (1.020–1.096)

Data are *P* percentiles, Univariate *OR* Odds Ratios and 95% *CI* Confidence Intervals. ^a^ *OR* odds ratio, computed for each 100 MET-min/wk.

## Main results

Table 2 shows the model built by multivariate binary logistic regression with the physical fitness, body composition, physical activity, health condition, and environmental hazards variables selected as the key risk factors/outcomes for falls, *p* < 0.05. The equation resulting from multivariate binary logistic regression modelling was the following:

$$ \pi (x)=\frac{esp\left(-0.053B-0.026L+0.027F-0.012 TPA+0.034 VPA+0.125R+0.112 HC+0.063H\right)}{1+ esp\left(-0.053B-0.026L+0.027 BFM\%-0.012 TPA+0.034 VPA+0.125R+0.112 HC+0.063H\right)} $$

Where π (x) is the probability of falling, exp. is exponential; B is multidimensional balance score (point); L is lean body mass (kg); F is fat body mass (%); TPA is total physical activity (100 METmin/wk); VPA is vigorous intensity physical activity (100 MET-min/wk); R is the Rest period (hr/day); HC is the number of health conditions; and H is the number of environmental hazards.

**Table 2:** Key risk factors for falling

Key risk factor	OR (95%CI)	Cutoff points	Specificity (%)	Sensitivity (%)	AUC (95%CI)
Multidimensional balance score (point)	0.949 (0.921–0.977)	0.250 0.359 0.500	0.341 0.640 0.873	0.860 0.694 0.349	0.710 (0.663–0.756)
Lean mass body mass (kg)	0.974 (0.956–0.993)				
Fat body mass (%)	1.027 (1.005–1.049)				
Total physical activity (100 MET-min/wk)	0.988 (0.977–0.999)				
Vigorous physical activity (100 MET-min/wk)	1.035 (1.013–1.057)				
Rest period weekdays (hr/day)	1.133 (1.016–1.264)				
Health conditions (n)	1.119 (1.038–1.206)				
Environmental hazards (n)	1.065 (1.021–1.111)				

Data are Multivariate *OR* Odds Ratios and 95% *CI* Confidence Intervals, MET Cutoff points for the probability of falling (π), Specificity, Sensibility, *AUC* Area Under the ROC Curve and 95% *CI*

The results illustrated in Table 2 show that for each additional point performed on the multidimensional balance score, the likelihood of falling decreases by 5.1%; for each additional kilo on lean mass body mass, the likelihood of falling drops by 2.6%; and for each additional 100 MET-min/wk. spent on total physical activity, the likelihood of falling decreases by 1.2%. For each additional 1% fat body mass, the likelihood of falling increases by 2.7% for each additional 100 MET-min/wk. spent on vigorous physical activity, the likelihood of falling increases by 3.5%; for each additional hr./day rest period weekdays, this likelihood increases by 13.3%; for each additional health condition, the likelihood of falling increases by 11.9%; and for each additional environmental hazard, this likelihood increases by 6.5%. These relationships are not dependent on age or sex since these two variables were not selected as significantly explaining the occurrence of falls in multivariate analysis. Finally, the model built by the multivariate analysis (and the respective equation) shows that a better outcome in one key risk factor may outweigh another weaker outcome on another key risk factor.

The Hosmer and Lemeshow goodness-of-fit test of the multivariate model was not significant (*p* = 0.985). The AUC was 0.710 (95% CI: 0.663–0.756), and the cutoff point maximizing specificity and sensitivity (64.0 and 69.4%, respectively) was 0.35939 (~ 35.9%). The AUC computed by cross-validation was 0.659 (CI 95%: 0.610–0.708).

**Fig. 1:**
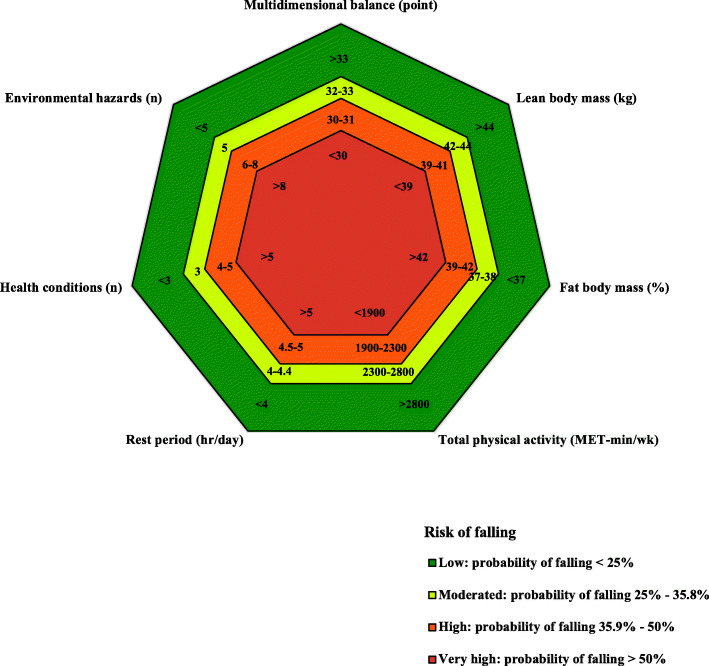
Cutoffs for a stratified risk of falling

Figure 1 illustrates the fall risk stratification according to the cutoff values computed for the outcomes of the key risk factors’ explaining fall occurrence (using multivariate binary regression modelling, ROC curves, and AUC analyses as explained in Methods).

Therefore, the variables on which an increase in the result corresponded to a decrease in the likelihood of falling, that is, multidimensional balance, lean mass body mass, and total physical activity - the cutoffs of π: 0.25, π: 0.35939 and π: 0.50 (used to stratify the risk of falling) corresponded to the 31st, 45th, and 58th percentiles, respectively. The variables on which an increase in the result corresponded to a decrease in the likelihood of falling, that is, fat body mass, vigorous physical activity, weekday rest period, health condition, and environmental hazards – the referred π cutoffs corresponded to the 69th, 55th, and 42nd percentiles, respectively. Illustrating these results, Fig. 1 shows the cutoff values and reference values usable for the stratification of fall risk in the studied population; they were as follows: multidimensional balance - low risk: score > 33, moderate risk: score 32–33, high risk: score 30–31, and very high: score < 30; lean body mass - low risk: > 44 kg, moderate risk: 42–44 kg, high risk: 39–41 kg, and very high: < 39 kg; fat body mass - low risk: < 37%, moderate risk: 37–38%, high risk: 39–42%, and very high: > 42%; total physical activity - low risk: > 2800 Met-min/wk., moderate risk: 2300–2800 Met-min/wk., high risk: 1900–2300 Met-min/wk., and very high: < 1900 Met-min/wk.; rest period weekdays - low risk: < 4 h/day, moderate risk: 4–4.4 h/day, high risk: 4.5–5 h/day, and very high: > 5 h/day; health condition - low risk: *n* < 3, moderate risk: *n* = 3, high risk: *n* = 4–5, and very high: *n* > 5; and environmental hazards - low risk: *n* < 5, moderate risk: *n* = 5, high risk: *n* = 6–8, and very high: *n* > 8.

Regarding the vigorous physical activity risk factor, as the 42nd, 55th, and 69th percentiles are equal to 0 MET-min/wk., the respective cutoffs and reference values are not shown in Fig. 1. Indeed, data showed that, concerning vigorous physical activity, only participants above the 80th percentile performed this kind of activity. The results of the multivariate binary regression showed that vigorous physical activity was positively associated with the risk of falling, especially when other key factors’ outcome results indicated high risk.

## Discussion

The present study assessed physical fitness, body composition, physical activity, health condition, and environmental hazard fall risk factors, aiming to identify the key risk factor outcomes and to establish their cutoff values for high-low risk. Hence, the present study established the outcome cutoffs and respective interval values for which the identified key risk factors indicate “low”, “moderate”, or “high” risk of falling, as recommended by the Centers for Disease Control and Prevention [32], or even “very high risk” of falling (see cutoffs and interval values on results). Such a stratified fall risk assessment approach in community-dwelling older adults was carried out using field tests that are older adults friendly and easily applicable to large communities.

The present study identified multidimensional balance, lean body mass, fat body mass, total physical activity, vigorous physical activity, weekday time in the rest seated position, health conditions, and environmental hazards as the key risk factors explaining fall occurrence. These results reinforce previous study findings [12, 15] by showing that poor results for physical fitness, body composition, and health condition and an increased number of environmental hazards are associated with an increased likelihood of falling. Regarding usual physical (in) activity, increased metabolic expenditure for total physical activity was associated with a decreased likelihood of falling. Likewise, increased inactivity - expressed as rest-seated periods on weekdays - was associated with an increased likelihood of falling. Hence, not only is low physical activity expenditure a serious risk for falls, as reported by others [33], but also is the daily time spent in inactivity, as evidenced by the present study. In addition, the present study found that engagement in vigorous physical activity was associated with an increased likelihood of falling. This result may seem controversial since other researchers [34] showed that the rate of incident falls in older community-dwelling ambulatory women was higher among those with low vigorous physical activity, excluding those with higher vigorous physical activity. However, it is important to note that the present study also observed that if the older adult was very fit and healthy (the typical nonfaller), the performance of vigorous physical activity was not a high riskily behavior for fall occurrence. In addition, as long as the older adults were less fit, unhealthier, and less prone to perform vigorous physical activity (the typical faller), the more riskily the performance of vigorous physical activity was.

The present study was the first to report the cutoffs and respective reference values for low, moderate, high and very high risk of falling for the included set of multidimensional key risk factors/outcomes, comprising physical fitness, body composition, physical activity, health condition, and environmental hazards (see cutoffs and interval values on results). The established cutoffs should be flags of alert for the risk of falling either for the older adult, either for caregivers or physicians or other health professionals, most likely because older adults and their caregivers frequently lack the perception of their fall risk [6, 7]. Moreover, physicians and other health professionals need outcome reference values for fall risk screening [8], as provided by the present study. For instance, it was shown that to avoid being at high risk of falling, older adults should score more than 32 points on the multidimensional balance test, have more than 41 kg on lean body mass, have less than 39% on fat body mass, spend more than 2300 MET-min/week on total physical activity, spend less than 4.5 h/day resting seated, have less than four health conditions and have less than five environmental hazards.

Previous studies have found different cutoff values for some of these key risk factors assessed by the same tests. For example, the study of Hernandez and Rose [17] computed a cutoff of 25 points distinguishing fallers from nonfallers through the multidimensional balance test results (analyzed single), and the study of Pereira and colleagues [27] computed a cutoff of 1125 MET-min/week distinguishing fallers from nonfallers in a younger and healthier population. In addition, there are other studies reporting cutoffs for different risk factors and outcomes, such as the time-up-and-go test and the four-square step test [14], and studies reporting specific health conditions, such as depression status, stroke, gouty arthritis, cataracts [35], sarcopenia and obesity [36, 37], as factors that determine the occurrence of falls. However, the present study was the first to examine the interaction between multiple risk factors to establish cutoffs and considered more risk stratification categories than previous studies, which might explain the different outcomes and cutoff values. To our knowledge, this approach is the first to allow easy establishment of a specific older adult’s fall risk profile.

Effective interventions require the management of the person’s identified risk threads and impairments, ascertained by a fall risk assessment [38]. The established cutoffs and respective interval values of low, moderate, high, and very high risk may be particularly valuable for designing effective approaches to fall prevention [15]. In particular, the established cutoffs enable quantification of how much each assessed older adult should improve on the identified risk factor/outcome to change from a higher risk level to a lower risk level of falling. For instance, consider an assessed older adult who achieves 29 points on multidimensional balance (classified as very high risk). Given that a score > 33 determines a low risk of falling, a score of 33–32 refers to a moderate risk, a score of 30–31 defines a high risk, and a score < 30 reports a very high risk of falling, the assessed older adult should improve his/her balance on 1 point to become at high risk, between 3 and 4 points to become at moderated risk, and a minimum of 5 points to become at low risk of falling. Similar reasoning can be applied to the other key risk factors’ outcomes. This approach will also facilitate posterior follow-ups [5] and the determination of the appropriateness of preventive measures to avoid falls.

Finally, the present study also found that a better outcome in one risk factor’s outcome may outweigh a weaker outcome in another risk factor. However, it must be highlighted that the amount of variation in one risk factor’s outcome necessary to promote a change from a higher risk to a lower risk level of falling is different from the necessary amount of variation in other risk factor’s outcomes. For example, while an increase of 5 points in multidimensional balance would be necessary to change from a very high risk of falling status to a low risk of falling status, the same change would require an increase of a minimum of 900 MET-min/week in physical activity. Moreover, it is important to note that the key risk factors reported by the present study are potentially modifiable and, as recommended, manageable through intervention [14]. However, the suitability of change is not the same for all risk factors [9, 15]. For example, while decreasing environmental hazards or increasing physical activity and improving balance may be relatively suitable for change, the same may not applicable for health conditions [9] (e.g., cataract surgery may cure visual impairment, but myocardial infarction has no cure).

The present study has some limitations. First, a faller was defined as a subject who had fallen at least once in the previous 12 months and not only as a subject presenting with recurrent falls as recommended by other researchers [39]. Nevertheless, the present study model explaining the occurrence of falls showed a capacity for discriminating fallers (AUC: 0.710, CI 95%: 0.663–0.756), similar to others focusing exclusively on recurrent fallers [40] (AUC: 0.710, CI 95%: 0.670–0.740), and the probabilities that were generated by the cross-validation procedure had a similar AUC (0.659, CI 95%: 0.610–0.708). Moreover, falls were assessed by a retrospective recall, which could be related to underestimation. Nevertheless, this retrospective estimation was considered acceptable, as falls’ assessment was obtained through an interview detailing the circumstances and the consequences regarding each fall. Second, an inherent limitation of the present study is its cross-sectional design, which does not allow establishing causality or directionality. For example, a poor balance or low total physical activity may increase the risk of falling, but a serious fall-related injury may also promote a loss of balance or decrease physical activity performance. Nonetheless, the error associated with the present cross-sectional study design was minimized by the inclusion of the criterion “absence of a recent health condition resulting in a temporary loss of physical fitness or dependence”, which ensured that physical fitness variables’ results did not meaningfully change in the previous year, and by the report of all participants’ physical activity maintenance over the previous year, as well as their health status, weight and environmental hazards. A similar approach was used in the study of Pereira et al. [27]. Third, to ensure data accuracy and participants’ independent living, the inclusion criterion of an absence of cognitive impairment was established, and therefore, this risk factor [19] was not included in the fall risk assessment. However, the gait pattern is a reported risk factor for falls [14], and future studies should examine its importance concerning the risk factors assessed by the present study. Nevertheless, the present findings should be accounted for when designing measures and programs for fall prevention in older adults. Both measures and programs should target quantitative improvements in detected impaired or risky outcomes and consider the identified cutoffs and respective interval values for fall risk stratification.

## Conclusions

This study identified multidimensional balance, lean body mass, fat body mass, total physical activity, vigorous physical activity, weekday time in the rest seated position, health conditions, and environmental hazards as key risk factors in fall risk assessment. Moreover, these outcome cutoffs and respective interval values for risk stratification as low, moderated, high, and very high risk were established (see values on results). Community-dwelling older adults’ fall risk assessments should focus on these outcomes in establishing individual older adults’ fall risk profiles; plus, the design of interventions for fall prevention should manage a person’s identified risks, taking into account the identified risk stratification cutoffs and respective interval values.

## Data Availability

The datasets used and/or analyzed during the current study are available from the corresponding author upon reasonable request.

## Abbreviations

CI: Confidence interval
FAB: Fullerton Advanced Balance
IPAQ: Physical Activity Questionnaire
MET: Metabolic equivalent of task
OR: Odds ratio
ROC: Receiver operating characteristic
AUC: Area under the curve
